# MANTRA game analytics: effectiveness of educational mobile game on knowledge gain and retention of Female Community Health Volunteers (FCHVs) and women in rural Nepal assessed through game analytics

**DOI:** 10.1186/s12889-026-26997-y

**Published:** 2026-03-19

**Authors:** Sonja Mueller, Katerina Stavrianaki, Andrei Boscor, Naomi Saville, Abriti Arjyal, Sushil C. Baral, Maureen Fordham, Gareth Hearn, Patty Kostkova

**Affiliations:** 1 Centre for Digital Public Health in Emergencies (dPHE), Department of Risk and Disaster Reduction, University College London, Gower Street, London, WC1E 6BT UK; 2 Department of Statistical Science, University College London, Torrington Place, London, WC1E 6BT UK; 3 Institute for Global Health, University College London, 30 Guilford Street, London, WC1N 1EH UK; 4 HERD International NP, Lalitpur, 44600 Nepal; 5 Centre for Gender and Disaster, Department of Risk and Disaster Reduction, University College London, Gower Street, London, WC1E 6BT UK; 6 Hearn GeoServe, Ltd, Worthing, UK; 7 Present Address: Centre for Sustainability, School of Geography, University of Otago, Dunedin, New Zealand

**Keywords:** Mobile Health (mHealth), Serious Games, Game Analytics, Maternal Health, Neonatal Health, LMICs

## Abstract

**Background:**

Mobile technology can deliver public health interventions to reach remote populations such as unique mHealth interventions aimed at low-literacy audiences in low resource settings. This research study assessed a mobile phone-based serious game that teaches geohazard, maternal, and neonatal health messages. This study is part of the Maternal and Neonatal Technologies in Rural Areas (MANTRA) project: Increasing maternal and child health resilience before, during, and after disasters using mobile technology in Nepal.

**Method:**

We develop a novel games analytics method assessing the knowledge gain and retention by the participants using the in-app collected session data of ordered player responses to game questions as they play and progress through the game. A full description of the study design can be found in (Mueller et al., BMC Public Health 20:1171, 2020). For each learning objective in each level we compare our observations (the data gathered from the players and coded as described above) and the expected frequencies (the number we would have seen as observed if the null hypothesis is true) with a chi – squared hypothesis test. Finally the test statistic is used to find the *p* value and compared to *p* = 0.05.

**Results:**

Knowledge gain and retention above 25% was calculated for 7 of 30 learning objectives. Results indicate only knowledge gained, so players answering correctly throughout the game are not the focus. In the maternal health module, a *p* value of < 0.05 was calculated for five learning objectives in level 1 and no significant learning objectives in level 2. The neonatal module level 1 had four learning objectives with significant results, and level 2 had three. The geohazards module showed the lowest significant results of the three modules, with only two significant learning objectives in level 2.

**Conclusions:**

Analyzing the MANTRA mobile health game data showed several successful learning objectives across the three modules of maternal health, neonatal health, and geohazards. Success implies the learning objectives and game help participants gain and retain knowledge, while other learning objectives can be targeted for redesign.

## Background

Mobile Health (mHealth) is a rapidly expanding area in health education and healthcare systems, and Kostkova succinctly presents the great potential of mHealth to impact the health education sector and future challenges facing the field [1]. mHealth initiatives on mobile devices have demonstrated value for overcoming obstacles of rough terrain, remote populations, and limited resources to distribute important public health information to hard to reach populations [2–5]. While a number of mHealth projects have been successfully implemented in clinical urban settings in LMIC [6, 7], mobile technology is of particular importance for health education and training for community workforce and local populations in rural and remote areas in low and middle income countries (LMIC).

MANTRA is one of the first mobile educational projects co-developed with local women in Nepal. The project, “Maternal and Neonatal Technologies in Rural Areas (MANTRA): Increasing maternal and child health resilience before during and after disasters using mobile technology in Nepal”, aimed to build women’s resilience by improving access to knowledge. This was done by developing mobile technology, a serious game, to support and expand existing participatory learning public health interventions and social protection mechanisms delivered by Female Community Health Volunteers (FCHVs). These volunteers typically serve small areas of about 1,000 people and run regular women’s groups.

Since its inception in 1988, the FCHV program has played a pivotal role in linking local communities with health related interventions [8]. One such intervention is MANTRA. The project worked with FCHVs to engage rural women and evaluated the impact of the game on both groups. The participants enjoyed the game and increased their awareness of danger signs while FCHVs viewed the app as a validation tool providing support for greater impact of their efforts in rural Nepal [9].

In rural and remote areas mHealth has the unique opportunity to deliver cost effective, easy to update/localise educational and training interventions [10, 11]. In many LMIC countries, primary health systems utilize health volunteers, who benefit from educational mHealth apps [11], low skilled and junior rural doctors in need for mobile decision support tools [12], and health and environmental agents responsible for community surveillance [13]. Evaluating these initiatives is a key step to demonstrate their value in achieving educational outcomes and appropriateness with the target audience in the health systems context.

This study aims to evaluate knowledge gain and retention from game analytics collected as women in Nepal played the MANTRA serious game. The game relies on players interpreting health conditions and risks from specifically designed pictograms of health conditions. The sequence of correct and incorrect answers as players move through increasingly difficult levels was logged in the application. Later analysis evaluated players’ knowledge gain and knowledge retention of the content in the game. This method of evaluation draws on game analytics to quantify the effectiveness of each learning objective to then improve the effectiveness of the game, thus delivering a comprehensive intervention to local rural communities.

Learning technology has made a major leap forward in the last decade. Serious and educational games, which are games with an educational purpose beyond entertainment, are now well established as a research domain [1, 14]. Learning effectiveness of serious games on learning has been demonstrated by knowledge assessment and evaluation [15–17]. However, how effective and impactful it actually is - remains the subject of ongoing research. In particular, knowledge assessment is an essential part of any educational experience, and serious games are no different [18].

Serious games are an important and as yet underutilized mHealth intervention opportunity to use game-based learning to supplement public health campaigns in LMIC settings [1, 19, 20]. Learning objectives are specific pieces of information or concepts within the module that support the broad educational and training project goals, these are a form of a syllabus in traditional training [21–24]. These objectives create a clear goal for the intervention that can be quantitatively evaluated. Assessing serious educational games by evaluating learning through knowledge assessment is essential to quantify an educational experience. Traditionally in serious games, pre- and post-play assessments of knowledge take place outside the game as a test or a survey, in line with teaching and intervention assessment methods [15, 16, 24, 25].

However, the focus on traditionally established pre- and post-assessment leaves the in-game collected interactive data and seamless assessment opportunities underused. In-app collected data provide an invaluable dataset for assessing performance and assessing user knowledge seamlessly without the participant knowing at every step (every click in fact) when interacting with the digital intervention [26]. The richness of the in-app collected dataset and insights these can bring through games analytics methods range better understanding of the impact of the mHealth educational intervention, feedback which parts of the games work and which did not work, leading to the opportunity to personalise the experience and learning process for each learner. These could have unprecedented impact if games analytics have been applied widely - so far these have not been much explored [26].

Taking a closer look at each player’s progression through a game allows the researcher a richer picture of the learning process, knowledge retention, and user interactions with the learning objectives and game itself. In support of the ultimate learning and knowledge retention goals of games-based learning, the usability, acceptability and cultural appropriateness of the game also impacts learning [27–29].

To improve the learning effectiveness, immediate feedback to the participant if the answer is correct or incorrect in the game itself to ‘reinforce learning’ has been proven to increase knowledge gain [30, 31]. Further building on immediate feedback, the ‘reinforced learning’ approach has been also built into educational tools as a successful integration strengthening the knowledge gain with immediate knowledge assessment [30, 31]. Reinforced learning has been proven to increase the knowledge gain [22, 24].

While MANTRA provides a unique example of such a successful educational mobile tool for low literacy workforce like FCHVs as well as rural women themselves, further research into the needs, challenges, and existing health systems in specific regions will ensure the technology is accepted by patients, health workers, communities, and healthcare systems [1, 32].

To study the effectiveness of the MANTRA serious game to retain health education knowledge, we developed a novel method to assess the impact of a serious game on FCHVs and rural women’s knowledge gain and retention using in-game collected session data and games analytics. This method evaluates the sequence of player responses collected in the game to construct a richer picture of the player experience and knowledge retention. To better understand how players gained and retained knowledge throughout the serious game intervention, we analysed how each player progressed through the game and the order of correct and incorrect responses for each learning objective. Analysing knowledge change and game-based learning identified successes and improvements is a novel approach easily generalizable for any mHealth education, training, or decision support systems, and will inform the next iteration of mobile games development, as well as insights that are transferable to similar research games projects in LMICs.

With these principles in mind, the interdisciplinary educational content of maternal, neonatal health, and geohazards were incorporated into MANTRA as a global health intervention. In the next section we illustrate on this unique study how a design of a successful game for such conditions could be evaluated using seamless evaluation and games analytics.

## Method

This evaluation method takes in game focuses on players’ knowledge retention so that the researcher can gain additional insights beyond a traditional pre- and post-test assessment. A brief outline of the game design, structure, and learning objectives is provided to aid in understanding the method of evaluation and results.

### MANTRA educational game design and learning objectives

Designing a serious game intervention consists of creating the structures needed for the game experience to achieve its training goals and subsequent evaluations, setting learning objectives based on the educational content, and choosing game mechanics appropriate for conveying the content. Contextualization and localization were vital concepts throughout the design process to tailor the content, artwork, animation, audio, and progression mechanisms to a specific culture and setting. These considerations appear in several mHealth studies in LMICs [2, 33, 34], and embedded cultural knowledge of in-country co-authors greatly aided the design process of the game MANTRA serious game [11]. The resulting learning objectives as text and corresponding pictogram are represented in [10].

The MANTRA game was built in Unity for Android, iOS, and Windows mobile devices (Unity Technologies, 2017). In field evaluations, the game was installed on Samsung Galaxy 7 phones to ensure proper functioning and consistency. After the field evaluations, game data related to player responses and user experience were recorded locally on the devices and later uploaded to a MongoDB back end server for later analysis (*The Most Popular Database for Modern Apps | MongoDB*, n.d.).

### Field tests set up and user demographics

Field testing took place in Kavrepalanchok District in Bagmati Province and suburban Kathmandu, in suitable villages identified by co-authors at Health and Social Development Forum (HERD) based on damage and visible geohazards related to the 2015 earthquake. Game analytics data was collected in field testing that took place in early November 2017, with 24 female participants recruited to the study through chain referral. Participant characteristics are summarized in Table 1. Smartphone ownership was of particular interest, and 42% of participants owned smartphones. The short field sessions result in some participants not beginning some modules or stopping in the middle of a module or level. Game analytics data was only recorded for 19 of these participants due to technical problems. The game content was consistent across the four testing sessions.

**Table 1:** Demographic characteristics of 24 participants from November 2017 field sessions

Characteristic	Group	*N*	% of Total Participants
Smartphone Ownership	Own smartphone	10	42%
	No smartphone	14	58%
Age	0–34 years	7	29%
	35 + years	17	71%
Education	0–4 years	8	33%
	5–9 years	3	13%
	10 + years	13	54%
Gender	Female	24	100%
Community Role	FCHV	19	79%
	Community Women	5	21%
Village	Chyamrangbesi	6	25%
	Chandenimandan	11	46%
	Imadol	7	29%

Field test sessions were facilitated by HERD and Nepal-based co-authors. First, the project was explained and informed consent obtained. Next, facilitators collected participants demographic information and experience with smartphones. Then, a pre-game test questionnaire of the 28 learning objectives to create baselines for the knowledge assessments was administered by a facilitator. Participants played the serious game for 10–30 min, with the app recording their responses and progress through the game. This was followed by a focus group discussion and a post-game test questionnaire identical to the first. Although facilitators advised participants to play individually, the players were in an open community space rather than laboratory testing conditions, so some discussed the game as they were playing.

### Dataset

The dataset analysed in this study is of ordered player responses to game questions as they play and progress through the game. Questions are formed by selecting a learning objective pictogram with its paired urgency pictogram, which will be the correct answer, then randomly selecting one to three other learning objectives of the opposite urgency or risk for the incorrect answer(s) depending on the level. There was always only one correct answer in all matching questions regardless of levels (one incorrect, to three incorrect were randomly displayed).

Once a question was successfully answered, the learning objective was not selected to be a correct answer again in that level as it was ‘tested’ and correctly answered (but is shown again in higher levels). If a question was answered incorrectly, the correct learning objective was added to an error pool and shown up to three times throughout the level. Therefore, the player will likely see the image multiple times in a level, both as the correct answer and as incorrect answers. The player continues the level until all learning objectives are seen and either a single learning objective is answered incorrectly three times and the player ‘fails’ the level, or the error pool contains only two learning objectives and the player ‘passes’ the level and progresses to the next level.

**Fig. 1:**
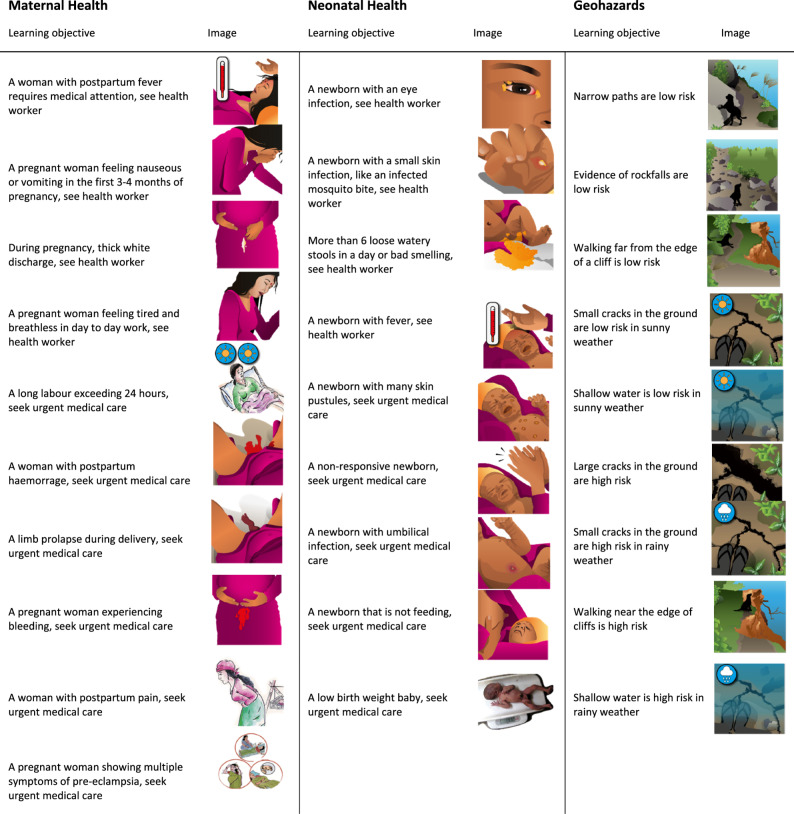
Learning objective pictograms for scenario component of questions. The image accompanying each learning objective is the designed image or placeholder image as designed for the second field test. Pictograms are categorized by module. Placeholder images are adapted from existing Mother and Infant Research Activities (MIRA) intervention materials and will be replaced in subsequent versions of the game (Source: MIRA )

The dataset is of players playing the game by answering multiple choice style questions composed of several pictograms from Fig. 1. Correct responses are coded *A*, and incorrect responses are coded *B*. An image can appear again if it was answered incorrectly up to a maximum of three times and therefore for each player, we obtain one of the following combinations: A, BA, BBA and BBB. In each module, responses are ordered by how players progress through the game, beginning with level 1 and if the level is passed, continuing to levels 2 and 3. Increasingly complex levels allow players to repeatedly see and interpret the learning objectives. It is important to note that this dataset is imperfect and may include incomplete codes, since participants were stopped in the middle of a level when the game play session ended.

### Statistical analysis

In this section we analyze the data from the game in a statistical way to assess the knowledge retention for each learning objective. The analysis is done at each level separately and for each learning objective we collect all the coded answers from the players. To assess whether there was knowledge gain and retention we perform a chi – squared (χ^2^ ) hypothesis test. The null hypothesis of the test is that the players answered randomly and the alternative hypothesis that the players did not pick the answers at random. Evidence against the null hypothesis i.e. the *p* value is less than 0.05 in the standard 0.5 level of significance (α), will demonstrate that the players used their knowledge to answer the questions. With the chi - squared test we compare our observations (the data gathered from the players and coded as described above) and the expected frequencies (the number we would have seen as observed if the null hypothesis is true). Since our null hypothesis is that the players chose the answers at random the corresponding probabilities for A, BA, BBA and BBB are 0.5, 0.25, 0.125 and 0.125. These probabilities are used to calculate the expected numbers. Following this the χ^2^ test statistic is calculated using the formula: ${\chi}^{2}={\sum}_{i}\frac{{({O}_{i}-{E}_{i})}^{2}}{{E}_{i}}$ where O_i_ are the observed data and E_i_ are the expected values under the null hypothesis. Finally the test statistic is used to find the *p* value.

When doing hypothesis testing the size of the effect that we are interested in capturing is important together with the sample size which are interlinked. To account for Type II errors we find the power of the test. Essentially a type II error means that an effect exists (the null is false) but we have failed to find it. Assuming a large effect, the power of the test is 0.42 due to the small sample size (*n* = 19). To achieve a power of 0.8 while keeping the effect size the same we would need a sample of 44 people. It should be noted that this is a unique dataset and there are both geographical and social barriers in obtaining a larger sample. Despite the small sample size we have demonstrated in some cases significant improvement in knowledge due to the game.

## Results

In this method, knowledge retention is demonstrated through the game by looking at the successive answers of each player throughout the different levels. If the user does not go back to answer “B” after correctly answering in a previous step then we can infer that the player learned the objective during the game. Players that have only answered correctly are excluded from the knowledge retention analysis as this can be interpreted as pre-existing knowledge. In the tables below we show the knowledge retention per learning objective in terms of absolute numbers and percentages. As the sample is small and some cases the players dropped out or accidentally restarted the module or game, listing both would be a better indicator.

In the maternity module (Table 2) the learning objective “A long labour exceeding 24 hours, seek urgent medical care” had the largest number of knowledge retention with a total of 9 players. For this learning objective 7 players answered correctly without making any mistake, 2 players returned to a wrong answer after replying correctly and 3 players replied solely incorrectly. The learning objectives “A pregnant woman feeling tired and breathless in day to day work, see health worker”, and “A limb prolapse during delivery, seek urgent medical care” showed that 0 players retained knowledge through the game. For learning objective “A pregnant woman feeling tired and breathless in day to day work, see health worker”, a very high number of players [11] replied correctly, 5 players returned to a wrong answer after replying correctly and 4 players answered solely incorrectly. From this we can infer there was a degree of pre-existing knowledge for this image. For “ A limb prolapse during delivery, seek urgent medical care”, 16 players answered correctly at all times, 2 players returned to a wrong answer after replying correctly and 2 players answered solely incorrectly showing a higher degree of pre-existing knowledge. The remainder of the learning objectives showed intermediate numbers of knowledge retention varying between 2 and 5.

**Table 2:** Maternal health module knowledge retention analysis results

Learning objective	Number of plays (all levels)	Total number of successes	Knowledge Gain & Retention (%)	Number of successes per level	Percentage of successes per level
A woman with postpartum fever requires medical attention, see health worker	62	29	25	Level 1: 15 Level 2: 8 Level 3: 6	Level 1: 42.86 Level 2: 44.44 Level 3: 66.66
A pregnant woman feeling nauseous or vomiting in the first 3–4 months of pregnancy, see health worker	52	32	15	Level 1: 18 Level 2: 8 Level 3: 6	Level 1: 64.29 Level 2: 44.44 Level 3: 100
During pregnancy, thick white discharge, see health worker	58	31	14.29	Level 1: 20 Level 2: 7 Level 3: 4	Level 1: 66.66 Level 2: 35 Level 3: 50
A pregnant woman feeling tired and breathless in day to day work, see health worker	51	30	0	Level 1: 16 Level 2: 9 Level 3: 5	Level 1: 55.17 Level 2: 60 Level 3: 71.43
A long labour exceeding 24 h, seek urgent medical care	57	31	42.85	Level 1: 16 Level 2: 10 Level 3: 5	Level 1: 42.10 Level 2: 76.92 Level 3: 83.33
A woman with postpartum haemorrage, seek urgent medical care	35	32	15	Level 1: 17 Level 2: 9 Level 3: 6	Level 1: 85 Level 2: 100 Level 3: 100
A limb prolapse during delivery, seek urgent medical care	43	33	0	Level 1: 18 Level 2: 9 Level 3: 6	Level 1: 75 Level 2: 69.23 Level 3: 100
A pregnant woman experiencing bleeding, seek urgent medical care	38	34	10	Level 1: 19 Level 2: 9 Level 3: 6	Level 1: 90.48 Level 2: 81.82 Level 3: 100
A woman with postpartum pain, seek urgent medical care	51	33	19.05	Level 1: 18 Level 2: 9 Level 3: 6	Level 1: 64.29 Level 2: 56.25 Level 3: 85.71
Vomiting or morning sickness	43	31	15	Level 1: 17 Level 2: 8 Level 3: 6	Level 1: 62.96 Level 2: 80 Level 3: 100
A pregnant woman showing multiple symptoms of pre-eclampsia, seek urgent medical care	49	32	9.52	Level 1: 19 Level 2: 7 Level 3: 6	Level 1: 65.38 Level 2: 41.17 Level 3: 100

The maternal module showed a high number of significant results. For level 1 the images with significant *p* values were: “A pregnant woman feeling tired and breathless in day to day work is less urgent, see health worker”, “A woman with postpartum haemorrhage requires urgent medical care”, “A prolapse during delivery requires urgent medical care”, “A pregnant woman experiencing bleeding requires urgent medical care” and “A pregnant woman showing multiple symptoms of pre-eclampsia requires urgent medical care”. In level two “A woman with postpartum haemorrhage requires urgent medical care” and “During pregnancy, thick white discharge is a minor danger. See health worker.” was close to being significant (*p* = 0.056). The high number of significant results in this module could be attributed both to the game but also to pre – existing knowledge.

The neonatal module (Table 3) also showed a high number of significant results in the chi squared test. In level 1, four images had *p* values < 0.05 namely “More than 6 loose watery stools in a day or bad smelling, see health worker”, “A newborn with many skin pustules, seek urgent medical care”, “A non-responsive newborn, seek urgent medical care” and “A low birth weight baby, seek urgent medical care”. In level 2, the statistically significant images are “More than 6 loose watery stools in a day or bad smelling, see health worker”, “A newborn with fever, see health worker”, and “A newborn with many skin pustules, seek urgent medical care”.

**Table 3:** Neonatal module knowledge retention analysis results

Learning objective	Number of plays (all levels)	Total number of successes	Knowledge Gain & Retention (%)	Number of successes per level	Percentage of successes per level
A newborn with an eye infection, see health worker	57	33	18.18	Level 1: 18 Level 2: 10 Level 3: 5	Level 1: 58.06 Level 2: 55.56 Level 3: 62.5
A newborn with a small skin infection, like an infected mosquito bite, see health worker	58	32	19.05	Level 1: 17 Level 2: 11 Level 3: 4	Level 1: 50 Level 2: 68.75 Level 3: 50
More than 6 loose watery stools in a day or bad smelling, see health worker	73	25	27.27	Level 1: 17 Level 2: 6 Level 3: 2	Level 1: 41.46 Level 2: 25 Level 3: 25
A newborn with fever, see health worker	69	29	28.57	Level 1: 19 Level 2: 6 Level 3: 4	Level 1: 55.88 Level 2: 24 Level 3: 57.14
A newborn with many skin pustules, seek urgent medical care	50	36	13.63	Level 1: 20 Level 2: 11 Level 3: 5	Level 1: 76.92 Level 2: 64.70 Level 3: 71.42
A non-responsive newborn, seek urgent medical care	50	34	4.55	Level 1: 18 Level 2: 11 Level 3: 5	Level 1: 66.67 Level 2: 64.70 Level 3: 83.33
A newborn with umbilical infection, seek urgent medical care	53	34	22.72	Level 1: 19 Level 2: 11 Level 3: 4	Level 1: 67.85 Level 2: 68.75 Level 3: 44.44
A newborn that is not feeding, seek urgent medical care	53	33	22.72	Level 1: 19 Level 2: 11 Level 3: 3	Level 1: 61.29 Level 2: 73.33 Level 3: 42.86
A newborn with hypothermia, seek urgent medical care.	62	28	13.64	Level 1: 16 Level 2: 9 Level 3: 3	Level 1: 50 Level 2: 42.86 Level 3: 33.33
A low birth weight baby, seek urgent medical care	45	38	9.52	Level 1: 21 Level 2: 12 Level 3: 5	Level 1: 87.50 Level 2: 75 Level 3: 100

The geohazards module showed the lowest number of significant results in the chi- squared test. In level 1 none of the images were significant and in level 2 only two images “Narrow paths are low risk”, and “Evidence of rockfalls are low risk”. The low level of significant results in the geohazards module demonstrates lack of pre-existing knowledge from the players. As such even small numbers in knowledge retention could be attributed to the game.

Table 2 is showing the results of knowledge retention for the maternity module. Players who answered solely correctly at all levels played (per learning objective) are excluded from this analysis as this can be interpreted as pre – existing knowledge. Five learning objectives had significant *p* values in level 1 and in level 2, two learning objectives were close to being significant. The maternity module showed the most significant results that can be attributed both to the game but also to their own knowledge.

Table 3 is showing the results of knowledge retention for the neonatal module. Players who answered solely correctly at all levels played (per learning objective) are excluded from this analysis as this can be interpreted as pre – existing knowledge. Zero learning objectives had significant *p* values in level 1 and two in level 2. The neonatal module also showed significant results that can be attributed both to the game but also to their own knowledge.

Table 4 is showing the results of knowledge retention for the geohazards module. Players who answered solely correctly at all levels played (per learning objective) are excluded from this analysis as this can be interpreted as pre – existing knowledge. Zero learning objectives had significant *p* values in level 1 and two in level 2. This was the most challenging module to the players and our results show low pre – existing knowledge. Having two significant learning objectives in level two can be attributed to knowledge obtained while playing the game.

**Table 4:** Geohazards module knowledge retention analysis results

Learning objective	Number of plays (all levels)	Total number of successes	Knowledge Gain & Retention (%)	Number of successes per level	Percentage of successes per level
Narrow paths are low risk	50	25	5.56	Level 1: 15 Level 2: 7 Level 3: 3	Level 1: 53.57 Level 2: 38.88 Level 3: 75
Evidence of rockfalls are low risk	58	24	5.88	Level 1: 15 Level 2: 6 Level 3: 3	Level 1: 57.69 Level 2: 27.27 Level 3: 30
Walking far from the edge of a cliff is low risk	49	26	11.11	Level 1: 15 Level 2: 7 Level 3: 4	Level 1: 60 Level 2: 35 Level 3: 100
Small cracks in the ground are low risk in sunny weather	39	27	29.41	Level 1: 15 Level 2: 9 Level 3: 3	Level 1: 68.18 Level 2: 64.29 Level 3: 100
Shallow water is low risk in sunny weather	46	28	35.29	Level 1: 14 Level 2: 11 Level 3: 3	Level 1: 50 Level 2: 91.67 Level 3: 50
Large cracks in the ground are high risk	44	28	17.64	Level 1: 14 Level 2: 10 Level 3: 4	Level 1: 58.33 Level 2: 66.67 Level 3: 80
Small cracks in the ground are high risk in rainy weather	45	27	11.76	Level 1: 15 Level 2: 8 Level 3: 4	Level 1: 75 Level 2: 40 Level 3: 80
Walking near the edge of cliffs is high risk	47	31	38.89	Level 1: 18 Level 2: 9 Level 3: 4	Level 1: 62.07 Level 2: 64.29 Level 3: 100
Shallow water is high risk in rainy weather	52	28	22.22	Level 1: 15 Level 2: 9 Level 3: 4	Level 1: 50 Level 2: 50 Level 3: 100

## Discussion

In this section we tackle several important points for discussion: linking the results across the MANTRA project; contextualizing the findings amongst other evaluations methods within the MANTRA project and similar studies, particularly focusing on the role of immediate feedback; the target audience of FCHVs and rural women; implications of the performance of individual learning objectives; limitations of the study; and future work.

### Comparing evaluation methods

The results of these analyses provide greater insight into the value of each knowledge assessment method presented in [11]. The paper-based knowledge assessments before and after the game play sessions treated the serious mobile game intervention as a ‘black box’. In this paper, we have used a new method to look deeper into the data recorded by the app of how participants progressed through the game to assess knowledge retention during the game play session. These two methods provide an opportunity to compare the methods and resulting insights.

To summarize, in terms of timeliness of data collection, the pre-survey on paper was administered first, then the participants played the game generating in-app data discussed in the section above, followed by post- survey administered on paper. A unique and important difference is that knowledge retention cannot be tested using simple pre- and post-assessment while the in-app games analytics session data allowed us to better understand whether players randomly answered correctly or whether they understood the learning objectives, retained the knowledge, and applied it in the higher levels.

We would like to highlight a few points about the game and field conditions. The game had a certain degree of randomness, such that in the short game play time, this may affect the visibility of learning objectives to participants and their knowledge from the game. We would like to highlight that in the paper-based pre- and post-assessments, participants were given no feedback on their selected answers, while in the game they received immediate feedback as positive or negative audio and visual cues. It was not practical to set up a single user study as the field sessions were held in open community spaces. Although participants were instructed to play individually, participants did talk with each other as they played. These practicalities and the value of such in-situ evaluations are detailed in [35], and for the MANTRA study specifically in [10].

As the researchers made sense of the data, several categories of ideal or typical response types recurred in our discussions illustrating some of the various pathways through the game for each learning objective. Categories or the simplified main ‘stories’ of players experiences were mainly based on the pattern of correct and incorrect answers for one player as they played through the levels of the game. The ideal learning case is where a participant answered incorrectly at the beginning of the game, but through the game levels they began answering the question correctly and continued to answer correctly through the remainder of the game including the paper-based post-assessment (ex. BAA or BBA). If the participant already has prior knowledge of the learning objective, then we would expect to see consistent correct answers throughout game play and paper-based assessment (ex. AAA). Participants that did not retain knowledge from the learning objectives during game play, would see many incorrect answers and perhaps a mix of correct and incorrect answers due to guessing (ex. ABB, ABA, or BAB).

There are many possible reasons for players diverging from these ideal scenarios. We considered that participants may have understood the learning objectives on paper and in the game differently. Such as: comparison questions of two or more learning objectives in the game were easier or challenging for participants to choose a relative risk, especially that the ‘questions’ posed in the two mediums were slightly different; switching between paper and mobiles confused players; and the presence of immediate feedback in the game and absence in the paper-based methods affected knowledge gain. Perhaps the game sessions were not long enough for players to take in all the learning objectives and with more time playing could gain and retain more knowledge. Another consideration is that while mobile technology may be more engaging for some players, others may find using a smartphone challenging, shifting their focus away from the learning objectives and toward the device itself.

Despite the differing performance based on the data above, in [9, 11] the authors explore the players’ experience of playing the game, which overall was quite positive, suggesting good engagement with the game, and an enjoyable learning experience.

Considering the knowledge retention analysis presented here, with other methods of evaluating the MANTRA serious game, shows the complexity of evaluating knowledge gain and learning. By exploring the experience through multiple methods, the researchers have a more complete, if more complex, picture and discussion of the mobile serious game’s effectiveness in providing health education messages to a low-literacy population. These promising, yet complex results indicate that future work for this serious game would be positively received by participants in the target audience, and should focus on another design, evaluation, and feedback cycle between researchers, designers, and participants to improve the game and evaluate its impact through a larger study, and if all goes well, eventual implementation.

### Immediate feedback to players

An important difference between the two methods was feedback to players. There was no feedback given after the paper based test to avoid bias in the data prior to the game play, while questions in the game incorporated immediate audio and visual positive or negative feedback after each pictogram move to reinforce learning. This immediate reinforcement supports the players’ knowledge progress through each step of the game. The knowledge retention and understanding of each pictogram condition is repeatedly tested throughout the increasing levels of difficulty leaving less space for ‘guess’ or accidental error. Looking at the game session as a whole, the paper-based assessment could be considered Level 0 as there was only one picture to assess, and from there, the in game assessment through questions become progressively harder from Levels 1 to 3.

While many learning objectives showed significant results in the two methods, some learning objectives only showed desirable results in one analysis, or neither analysis. Comparing module results from the two knowledge measurements, the geohazards module as a whole showed the greatest knowledge gain of the three modules in both measurements probably due to the lower prior knowledge of the subject. Maternal and neonatal health modules showed some knowledge gain in both measurements, although both modules had higher measures of prior knowledge than the geohazards module [36].

### Target audience challenges

The target audience for this intervention, rural women and FCHVs with low literacy, living in hardly accessible remote rural villages, presented many cultural and geographical challenges. Some women and FCHVs have to walk on foot through hilly rough terrain for up to two hours to attend the monthly women group sessions. While this is a challenge for implementing public health, educational and training programmes, it also provides an opportunity highlighting how revolutionary mHealth interventions are for workforce training and primary health education in rural communities. Since many challenges in reaching this target audience were relevant in the analysis presented in [36] this sub-section will reiterate these points which are vital to contextualizing the knowledge retention analyses in the setting.

Throughout the sessions, participants wanted to play together and seemed to enjoy the social atmosphere. Playing together could remove some barriers and reduce reinforcing existing vulnerabilities such as smartphone skills and ownership, as well as encourage discussion and awareness of maternal and neonatal health and geohazard issues. Although both FCHVs and women participants enjoyed playing the games in facilitated sessions among their peers, would they continue playing at home for educational and training reasons when busy with their households? Another opportunity of using the educational game at home may bring women a source of empowerment by citing the game to negotiate healthcare access in traditional Nepali patriarchal households, where husbands and mothers in law often make decisions regarding healthcare [37, 38]. The research team recognized the potential to reach a larger audience than our target audience and the importance of testing their acceptance of the intervention. For this reason, one field session (not included in the game analytics data of this study) consisted of younger men who may have families which could be impacted by the conditions illustrated in the intervention which also brought positive results in terms of knowledge gain and general feedback.

Another challenge to reaching the target audience was the rural locations and steep topography. Much of rural Nepal is remote and hilly or mountainous, and transportation is infrequent, time consuming, and weather dependent, so access to many rural villages is difficult and journeys may take hours or days. Some potential participants from the stages of the study living on the edges of the community were unable to attend follow-up sessions due to domestic responsibilities and travel times/distance across the challenging terrain.

In the future, using the mHealth tools from home to communicating with the women community and FCHVs as and when needed, rather than only once a month at the group sessions, would strengthen the educational impact of the intervention and create a sense of community and group support between FCHVs and rural women in the area. Also across different regions in Nepal which is impossible at the moment due to travel difficulties, cost and geographical terrain barriers.

A recent study [39] also looks at the implementation of mHealth interventions in Nepal, focusing on improving the health communication skills of FCHVs and their beneficiaries. This qualitative study also demonstrated the effectiveness of mHealth interventions in LMICs.

Since we focused our data collection amongst FCHVs and reproductive aged women, for whom the mobile game was devised, our sample is not representative of the general Nepalese population. Our sample size is small, yet we were able to evaluate the serious game on a small scale to support the next phase of the project at a larger scale implementing the intervention across Nepal and in other LMICs where primary care is delivered through a similar rural workforce such as Nepal’s FCHVs. We were also able to contextualise statistical results through discussions and observations of game play in a real-world setting, rather than laboratory conditions, to “understand how technology is and can be used in the everyday real world, in order to gain new insights” regarding engagement, impacts, and behavior when faced with a new technology [35].

### Interpreting performance of specific learning objectives

The high percentages and correct answers in the more difficult later levels is evidence that learning occurred later in the game when there were fewer players reaching level 2 in each module, and even fewer reaching level 3, so the small size of the datasets only suggests this possibility rather than robustly supporting it. Another factor could be that participants took longer to understand the unfamiliar geohazards rather than familiar health topics regularly taught by FCHVs, including previous picture cards from Mother and Infant Research Activities [24, 31, 40–42].

Learning objectives that performed poorly need improvement to clearly convey the intended message. Identifying specific learning objectives in need of improvement allows the research team to use resources efficiently by prioritizing redesign of specific learning objectives in subsequent iteration of the game.

New topic modules with lower baseline knowledge may produce knowledge gain assessment data that looks more like the geohazards module, with greater knowledge gain or change within the target audience as there was a higher opportunity for knowledge improvement than in the maternal and neonatal health modules, with which FCHVs and women were more familiar. Thus, more advanced topics in these vital areas would be developed in the future versions of the training tool.

### Limitations

Testing long term retention of the concepts presented in the serious game was not practical in the short time frame and limited resources of the project. Additionally, FCHVs’ regular engagement with health topics would interfere with measuring knowledge retention from the intervention in the long term.

Measuring the translation of knowledge to health seeking behavior is very challenging and resource intensive [9]. Producing behavior change is the ultimate purpose of the serious game intervention, either through personal decisions or negotiating with family decision-makers. Continued engagement with the intervention could be challenging, as independent use without research facilitators could create higher demands of users’ phone skills to use the game and troubleshoot technical issues. Also, would they use the game for advice when they or their child suffered a condition taught as a learning objective? The answer to this question would influence their healthcare seeking behavior at the point of need. Home use of the mobile serious game may bring women a source of empowerment by citing the game to negotiate healthcare access in their households, where husbands and mothers in law often make decisions, including regarding healthcare [38].

The MANTRA pilot study was conducted in open communal spaces within the study locations where rigorous test conditions were not possible. Variables within our control were providing the game on similar Android mobile phones brought by the study facilitators and requesting participants played alone. Some technology problems were addressed “on the fly”, yet the minor changes made during the few days of the field tests did not substantially change the game across the field test sessions presented in this paper. The research team approached the field tests as in situ studies or research in the wild to gain insight rather than rigorous data collection expected in a lab experiment [35].

Scaling up a pilot intervention for wider testing and eventually public distribution could take several paths and involves many challenges. Involving a Nepalese game developer could accelerate the process of further developing and distributing the serious game intervention. A potential intermediate step for scaling up is training FCHVs to incorporate the serious game into existing community workshop programs. Distribution is a challenge, with incomplete mobile network coverage a barrier to obtaining and updating the game files. One solution might be to allow the game files to be shared from phone to phone via Bluetooth or a wired connection, although this has its own challenges. Interoperability between available hardware devices is another obstacle.

While the app requests smartphones which are not widely affordable in Nepal at the moment, the projection of mobile app availability and ownership is growing [9] and it is therefore reasonable to assume in a very near future address to smart phones will not be an issue for local rural women. The game runs locally on the phone and does not require network connectivity, although, as a sustainable intervention updates and new versions could be installed when the user has Wi-Fi or data connectivity.

During field tests and evaluation, facilities were open community spaces. This setting posed difficulties for implementing a single user study with rigorous test conditions since participants played in groups and chatted. This challenge is well known among in situ and semi-structured qualitative studies [28, 35]. Although rigorous user testing was not possible, the in situ testing provided valuable insights of how the intervention would actually be used in a facilitated educational group or as a stand-alone game to play at home.

### Future work

Building on this project and looking to the next steps, there is potential to reach and impact the target audience as well as additional groups, explore possibilities to scale up the global health intervention, and effectively involve project partners and stakeholders in the co-design process.

Exploring educational games as global health interventions would build evidence and best practice to overcome barriers and deliver vital health information. Future field evaluations in a larger and more diverse population sample with randomly selected participants would both investigate the effectiveness of the intervention and the potential to scale up the intervention, perhaps with additional health related topics such as nutrition or vaccination. Similarly, this method to analyze knowledge retention could be applied in these other modules as well as other interventions to see how players retain knowledge through other serious games. Producing behavior change is the ultimate purpose of the serious mobile game intervention, which remains a challenging open research question for a longitudinal follow up study with project partners and stakeholders.

Looking forward, continuing development of the MANTRA serious mobile game could provide further evidence to support serious games as an effective delivery method in the field of mHealth. For the MANTRA serious mobile game specifically, there are several areas to continue developing the intervention. Firstly, the feedback and results of evaluations should be considered for feasibility and incorporated into the serious mobile game, such as animated pictograms or audio instructions. There is potential to explore the app functionality beyond education to include communication with healthcare facilities, such as sending a photograph of ailments or calling an ambulance, and crowd-sourced reporting of hazards impeding road transport so authorities can plan better routes to deliver emergency medical services. Thirdly, support for interaction between FCHVs across the country and between rural women and FCHVs would be a much needed empowered of rural women, also widening the application of learned skills of FCHVs.

Additionally in the next stages, the project could be expanded to cover new topics such as nutrition (which was suggested by the FCHVs) and other LMIC countries (by localising the artwork), and to deploy in LMIC countries where health education of rural population is limited. Finally, scaling up a pilot intervention for wider testing and eventually public distribution could take several paths and would involve challenges a long term, large scale training programme could deliver. A potential intermediate step for scaling up is training FCHVs to incorporate the serious game into existing community workshop programs.

## Conclusions

Training rural workforces and educating women in communities about maternal and neonatal health is of major importance for people living in remote areas in LMIC settings. Mobile technologies have an unprecedented potential to deliver training and improve health knowledge, awareness of high-risk conditions and healthcare seeking behavior in rural settings. The MANTRA project has developed a mobile educational game improving knowledge of maternal health, neonatal health and geohazards in workforce FCHVs and community women in rural Nepal, and demonstrated significant knowledge gain.

This study of the effectiveness of the MANTRA serious game provides an example and method to evaluate potential mHealth interventions which use games-based learning as a tool to educate low literacy audiences amidst rapidly increasing accessibility of digital technology in low income settings. Serious games are the ideal tool to deliver targeted training and educational interventions in rural settings and provide unique insights into the participants’ knowledge gain and retention through game analytics of the in app collected session data. A novel analytics method was described in this study to assess knowledge improvement throughout the game, rather than traditionally before and after the intervention. This unique method was applied on the MANTRA mobile health game data to highlight the most successful learning objectives for knowledge retention in the three modules - maternal, neonatal and geohazards, and compared with the traditional pre- and post-knowledge assessments. This novel knowledge assessment methodology is generalisable to any educational games interventions and should be deployed to improve and personalise the training tools.

This project has demonstrated the potential of a serious game co-developed with local users in LMIC to improve their health knowledge. The intervention is easy to scale up and localisable to other conditions and contexts, and provides a blueprint for a development of such interventions for LMICs.

## Data Availability

The datasets used and/or analyzed during the current study are available from the corresponding author on reasonable request.

## Abbreviations

FCHV: Female community health volunteers
LMICs: Low and middle income countries
LO: Learning objectives
MANTRA: Maternal and Neonatal Technologies in Rural Areas
mHealth: Mobile health
